# GATA4 as a novel regulator involved in the development of the neural crest and craniofacial skeleton via Barx1

**DOI:** 10.1038/s41418-018-0083-x

**Published:** 2018-03-09

**Authors:** Shuyu Guo, Yuxin Zhang, Tingting Zhou, Dongyue Wang, Yajuan Weng, Qi Chen, Junqing Ma, Yi-ping Li, Lin Wang

**Affiliations:** 1 Jiangsu Key Laboratory of Oral Diseases, Nanjing Medical University, 140 Hanzhong Road, Nanjing, 210029 China; 2 Atherosclerosis Research Center, Key Laboratory of Cardiovascular Disease and Molecular Intervention, Nanjing Medical University, 140 Hanzhong Road, Nanjing, 210029 China; 3 Department of Pathology, University of Alabama at Birmingham, SHEL 810, 1825 University Boulevard, Birmingham, AL 35294-2182 USA

## Abstract

The role of GATA-binding protein 4 (GATA4) in neural crest cells (NCCs) is poorly defined. Here we showed that mouse NCCs lacking GATA4 exhibited developmental defects in craniofacial bone, teeth, and heart. The defects likely occurred due to decreased cell proliferation at the developmental stage. The in vitro results were consistent with the mouse model. The isobaric tags for relative and absolute quantitation assay revealed that BARX1 is one of the differentially expressed proteins after GATA4 knockdown in NCCs. On the basis of the results of dual-luciferase, electro-mobility shift, and chromatin immunoprecipitation assays, *Barx1* expression is directly regulated by GATA4 in NCCs. In zebrafish, *gata4* knockdown affects the development of NCCs derivatives. However, the phenotype in zebrafish could be partly rescued by co-injection of *gata4* morpholino oligomers and *barx1* mRNA. This study identified new downstream targets of GATA4 in NCCs and uncovered additional evidence of the complex regulatory functions of GATA4 in NCC development.

## Introduction

GATA-binding protein 4 (GATA4) is a transcription factor that has been extensively studied in heart development and has multiple essential roles in cell proliferation, survival, and differentiation [1]. Depletion of GATA4 in the mouse embryos leads to lethal cardiovascular defects [2, 3]. Moreover, knockdown of *gata4* expression in zebrafish by morpholinos (MO) resulted in an unlooped heart tube [4], suggesting that GATA4 has a conserved role in heart development from fish to mammals.

The neural crest (NC) is a multipotent cell population that originates from the dorsal neural tube and gives rise to numerous cell types [5]. Defective NC development is usually associated with many congenital birth abnormalities, including syndromes such as DiGeorge, Treacher–Collins, CHARGE, and Hirschsprung’s disease [6–8]. The development of neural crest cells (NCCs) can be broadly divided into three stages: formation, migration, and differentiation [9]. The inadequate development of NCCs during these stages may result in any of the aforementioned syndromes. The NC derivatives include bone and cartilage of the skull as well as tendons, muscles, and connective tissues of the ear, eye, teeth, and heart [5]. Due to their remarkable plasticity, the development of NCCs requires a complex landscape of transcriptional control [10].

It has been reported that GATA4 is widely expressed in developing embryos [11]. Early GATA4 expression is detected in intra-embryonic cells of the blastocyst. Moreover, GATA4 is also expressed within migratory NCCs. The specific NCCs marker *Sox10* is co-expressed with GATA4 in a large subset of cranial NCCs during embryo development [11]. Our previous microarray data (unpublished data) showed that the GATA4 expression level in the mouse maxillofacial tissues was significantly higher at embryonic day 13.5 (E13.5) than at E18.5. This may indicate that GATA4 plays an essential role in NCCs during the embryo maxillofacial development stage. Moreover, GATA4 also functions as a pioneer factor in osteoblasts [12] and is vital for bone mineralization [13]. These findings prompted our interest in exploring whether GATA4 is involved in the development of NCC derivatives. Although great progress has been made toward understanding the gene regulatory networks underlying NC development, the function of GATA4 in NC development remains poorly understood.

To date, there have been no reports on the specific mechanism of GATA4’s role in NCC development due to the early lethality of GATA4-knockout embryos at around E8.5–E9.5 [14, 15]. Therefore, conditional loss-of-function models for GATA4 genes could be applied to reveal the role of GATA4 in NCCs. In this study, we generated and examined the *Wnt1-Cre;Gata4*^*fl/fl*^ conditional knockout mouse model in which GATA4 was conditionally ablated in the NCCs. In addition, the phenotype associated with MO-induced knockdown of *gata4* in zebrafish was assessed and observed to be consistent with that obtained in mice. Taken together, these results obtained in the two independent animal models described herein offer interesting new insights into the key role of GATA4 in regulating NC development.

## Results

### Mutant mice exhibit craniofacial and cardiac defects

**Fig. 1:**
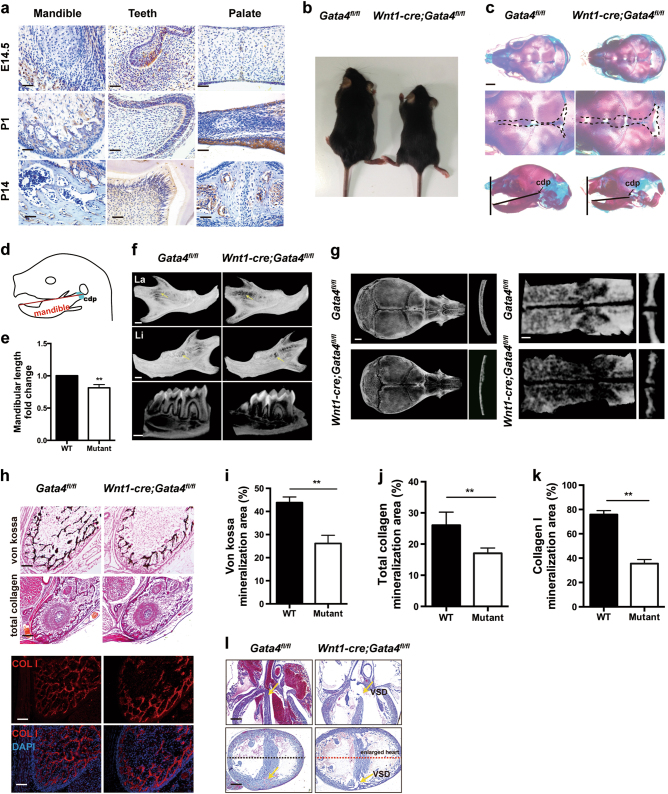
Mice lacking GATA4 in NCC-derived tissue exhibit craniofacial and cardiac defects. **a** Localization of GATA4 expression during mouse development at embryonic day 14.5 (E14.5) and at postnatal day 1 (P1) and P14 (scale bar: 50 μm). Brown staining corresponds to anti-GATA4 immunoreactivity. **b** Photographic analysis of 3-week-old *Wnt1-cre;Gata4*^*fl/fl*^ (Mutant) mice and *Gata4*^*fl/fl*^ (WT) mice (*n* = 5). (**c**, Upper) *Wnt1-cre;Gata4*^*fl/fl*^ and *Gata4*^*fl/fl*^ P1 skulls stained with Alizarin red and Alcian blue. (**c**, Middle) Magnification of the calvaria with widened cranial frontal sutures in mutant mice. (**c**, Lower) Sagittal view shows the mandibular retrognathism of the mutant mice. Scale bar, 500 μm. **d**, **e** Quantitative analysis of the length of the mandible (*n* = 5). **f** Three-dimensional micro-CT image of the mandible and teeth at P21. Mutant mice displayed a drastic decrease in mineralization and short root abnormalities. Yellow arrow indicates the decreased mineralization area. Scale bar, 500 μm. **g** Three-dimensional micro-CT image of the skull (Left) and palate bone (Right) at P21. The right pictures of each group show the thickness of the frontal bone and palate bone. **h** Mandible tissue stains of von Kossa, total collagen, and Collagen I at P1; scale bars: 100, 200, and 100 μm, respectively. **i**–**k** Quantitative analysis of the positive area (the whole image section was analyzed for the mineralization area quantification) of von Kossa, total collagen, and Collagen I staining, respectively (*n* = 5). **l** Sagittal and coronal view of the heart in mutant mice showed VSD and enlarged hearts; scale bar: 200 μm. The data are presented as the mean ± S.E.M. from at least three independent experiments. **P < 0.01. cdp condylar process, CT computed tomography, la labial, li lingual, NCC neural crest cell, VSD ventricular septal defect

According to a previous study [11], GATA4 transcripts are found within most of the migratory NCCs. Thus, we examined the expression patterns of GATA4 in the mouse NCC-derived craniofacial tissue using immunohistochemical analysis at E14.5, postnatal day 1 (P1), and P14 (Fig. 1a; detailed descriptions and magnified images of each panel can be found in the Supplementary Figure S1a–c). In the mandible, GATA4 was expressed in the osteoblasts, and the positively stained cells were distributed along the surface of the bone trabecular (Supplementary Figure S1a). In the teeth, GATA4 was expressed in the dental mesenchyme, odontoblasts, and ameloblasts (Supplementary Figure S1b, red arrows, black arrows, and yellow arrows, respectively). In the palate, GATA4 was expressed both in the palate epithelium and palate mesenchyme at E14.5 and P1. In the palate at P14, the GATA4-positive cells (osteoblasts) were distributed along the surface of bone trabecular (Supplementary Figure S1c).

To investigate whether GATA4 controls craniofacial development, we ablated GATA4, specifically in NCCs and NCC-derived tissues, by crossing a floxed *Gata4* allele with a *Wnt1-Cre* driver. The *Wnt1-cre;Gata4*^*fl/fl*^ (mutant) mice were born in the predicted Mendelian ratio and survived into adulthood. Compared with control *Gata4*^*fl/fl*^ (wild-type, WT) littermates, 3-week-old *Wnt1-cre;Gata4*^*fl/fl*^ mutants exhibited short stature (Fig. 1b and Supplementary Figures S1d–f). We subsequently performed Alcian Blue and Alizarin Red staining at P1 to examine the role of GATA4 in cranial development. On the basis of the results, the mutants exhibited widened cranial frontal sutures and mandibular retrognathism (Fig. 1c–e). Micro-computed tomography (micro-CT) scanning analysis revealed a drastic decrease in mineralization of the mandible, frontal bone, and palate in mutants (Fig. 1f and g and Supplementary Figure S1g–j). In addition, the mutants’ teeth were grossly smaller than those of controls (Fig. 1f). Von Kossa, total collagen, and collagen I staining revealed a substantial decrease in mineralization in the mandible of P1 *Wnt1-cre;Gata4*^*fl/fl*^ mice compared with that in control mice (Fig. 1h–k). Strikingly, the absence of GATA4 in NCCs induced severe cardiac defects with a prominent ventricular septal defect (VSD) and enlarged heart (Fig. 1l). However, cardiac development was beyond the scope of the present study, which focused on craniofacial development.

### Lack of GATA4 in NCCs affects proliferation at E14.5

**Fig. 2:**
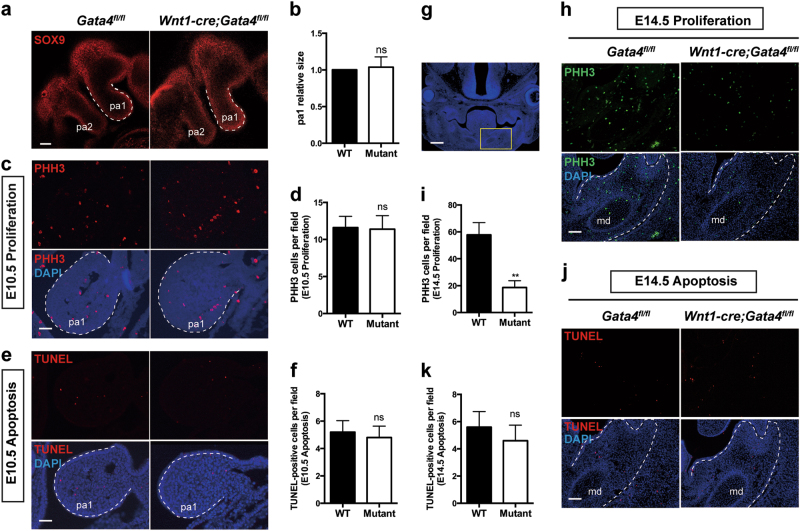
Loss of GATA4 from NCC-derived tissue reduces cell proliferation in mandible at developmental stage. **a** *Gata4*^*fl/fl*^ (WT) and *Wnt1-cre;Gata4*^*fl/fl*^ (Mutant) embryos at embryonic day 9.5 (E9.5) were stained with SOX9 antibody, a marker of early migrating NCCs. There were no significant differences between the two groups. The dotted line shows the pa1; scale bar: 200 μm. **b** Quantitative analysis of the size between the two groups (*n* = 5). **c** Sagittal sections through pa1 in E10.5 *Gata4*^*fl/fl*^ and *Wnt1-cre;Gata4*^*fl/fl*^ embryos. Sections were immunostained with PHH3 (red) for cell proliferation and counterstained with DAPI (blue); scale bar: 50 μm. **d** Quantitative analysis of cell proliferation in pa1 (*n* = 5). **e** Sagittal sections through pa1 in E10.5 *Gata4*^*fl/fl*^ and *Wnt1-cre;Gata4*^*fl/fl*^ embryos. Sections were immunostained with TUNEL (red) for cell death and counterstained with DAPI (blue); scale bar: 50 μm. **f** Quantitative analysis of cell death in pa1 (*n* = 5). **g** Whole skull sections are shown, and the yellow insert in the section indicates the embryo diagram to the right; scale bar: 200 μm. **h** Coronal sections of the head in E14.5 *Gata4*^*fl/fl*^ and *Wnt1-cre;Gata4*^*fl/fl*^ embryos. Sections showed the mandibular area immunostained with markers of cell proliferation, PHH3 (green), and counterstained with DAPI (blue); scale bar: 100 μm. **i** Quantitative analysis of cell proliferation in the mandible (*n* = 5). **j** Coronal sections of the head in E14.5 *Gata4*^*fl/fl*^ and *Wnt1-cre;Gata4*^*fl/fl*^ embryos. Sections showed the mandibular area immunostained with TUNEL (red) and counterstained with DAPI (blue); scale bar: 100 μm. **k** Quantitative analysis of cell death in the mandible (*n* = 5). The data are presented as the mean ± S.E.M. from at least three independent experiments. ^**^P < 0.01. DAPI 4,6-diamidino-2-phenylindole, NCC neural crest cell, md mandible, ns not significant, pa pharyngeal arch, PHH3 phosphohistone H3, TUNEL terminal deoxynucleotidyl transferase dUTP nick end labeling

Given that the loss of GATA4 in NCCs impaired both bone and tooth growth during development (Fig. 1), we hypothesized that GATA4 may have a direct effect on early NCC development, in addition to its roles in migratory NCC [11]. To validate our hypothesis, we analyzed the pharyngeal arch 1 (pa1) by staining for SOX9, a marker of early migrating NCCs, in E9.5 embryos. Our results showed no differences in NCC migration into the pa1 between WT and mutant mice (Fig. 2a, b). Additionally, phosphohistone H3 (PHH3) and terminal deoxynucleotidyl transferase dUTP nick end labeling (TUNEL) staining at E10.5 also showed no significant differences in cell proliferation and death between *Wnt1-cre;Gata4*^*fl/fl*^ and WT mice (Fig. 2c–f). Therefore, these results suggested that the deletion of GATA4 had no effect on NCC migration into pa1. Instead, phenotypic changes due to GATA4 conditional knockout were likely to occur at a later developmental stage. Thus, we performed the PHH3 and TUNEL staining in E14.5 embryos at the mandibular area (Fig. 2g). The results showed that the specific loss of GATA4 in NCCs decreased cell proliferation in the mandible (Fig. 2h, i); however, the apoptosis levels were comparable between mutant and WT mice (Fig. 2j, k). Taken together, these observations suggest that GATA4 is essential for the development of NCC-derived craniomaxillofacial derivatives through its role in promoting proliferation at the developmental stage.

### GATA4 regulates the function of NCCs in vitro

**Fig. 3:**
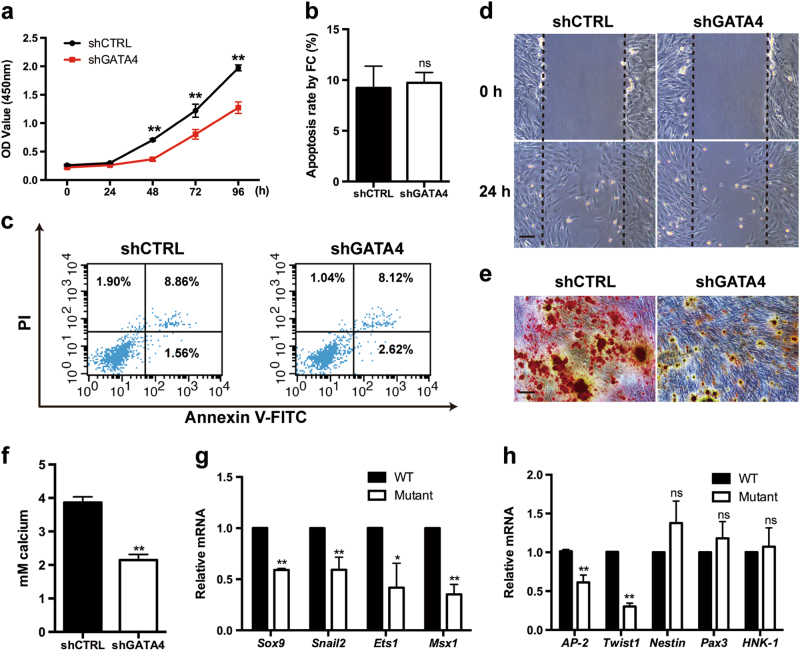
Inactivation of GATA4 in NCCs impairs cell proliferation, migration, and differentiation in vitro. **a** Cell counting kit-8 assay was used to examine the proliferation of NCCs after infection with GATA4 lentivirus (*n* = 5). **b**, **c** Quantitative analysis of cell death by flow cytometry showed no significant difference between the two groups (*n* = 5). **d** Wound scratch assay indicated attenuated migration ability of NCCs at 24 h after GATA4 knockdown; scale bar: 100 μm. **e** After NCCs mineralization for 14 days, Alizarin Red staining was conducted and observed under microscope; scale bar: 100 μm. **f** Semi-quantitative estimation of calcium (*n* = 5). **g**, **h** The qRT-PCR assay indicated downregulation of NCCs key genes in the pa1 of embryos at embryonic day 10.5 (E10.5) after GATA4 knockout in NCCs (*n* = 5). The data are presented as the mean ± S.E.M. from at least three independent experiments. ^*^P < 0.05, ^**^P < 0.01. NCC neural crest cell, ns not significant, pa1 pharyngeal arch 1, qRT-PCR quantitative reverse transcription-polymerase chain reaction

To provide further evidence of the function of GATA4 in vitro, GATA4 expression was reduced in primary embryonic NCCs collected from adult WT mice by using a small hairpin RNA (shRNA)-mediated knockdown approach. The results indicated that GATA4 knockdown decreased the proliferative ability of NCCs, as measured by cell counting kit-8 (CCK-8) assay, compared with that of the control group on culture day 4 (Fig. 3a). However, Annexin V-fluorescein isothiocyanate (FITC)/propidium iodide staining showed no significant difference in the percentages of apoptotic cells between the two groups (Fig. 3b, c). Moreover, shGATA4-treated NCCs exhibited slower scratch closure, indicating that the migratory ability was attenuated (Fig. 3d). The osteogenic differentiation ability of NCCs treated with shGATA4 followed by mineralization for 14 days was significantly decreased compared with the control group (Fig. 3e, f). Furthermore, we extracted the mRNA from the pa1 of WT and mutant mice at E10.5 and performed real-time quantitative polymerase chain reaction (qPCR) analysis for nine key genes of interest including *Sox9*, *Snail2*, *Ets1*, *Msx1*, *AP-2*, *Twist*, *Nestin*, *Pax3*, and *HNK-1*, which are known to play important roles in the development of NCCs. As demonstrated in Fig. 3g,h, the mRNA expression levels of these key genes (except *Nestin*, *Pax3*, and *HNK-1*) were decreased dramatically after GATA4 knockdown. Taken together, these in vitro findings are consistent with the in vivo observations (animal model phenotype) and the hypothesis that GATA4 is critical for the function of NCCs.

### BARX1 as a distinct protein identified by isobaric tags for relative and absolute quantitation (iTRAQ) analysis in NCCs after GATA4 knockdown

**Fig. 4:**
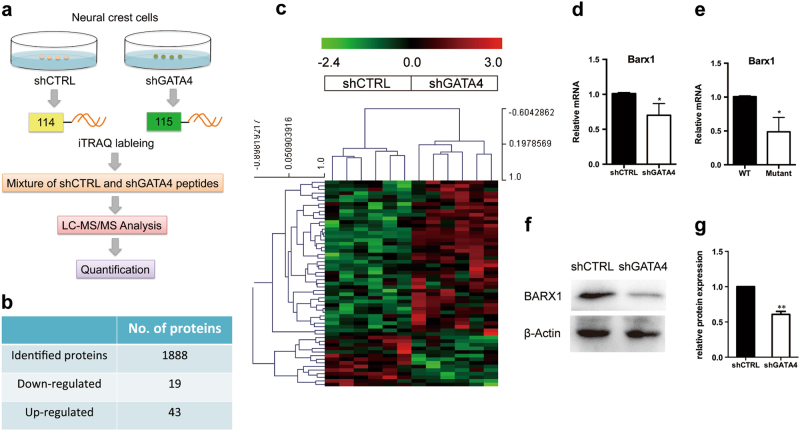
BARX1 is one of the differential proteins identified in NCCs after GATA4 knockdown by iTRAQ assay. **a** Flow chart of proteomic analysis for identification of differentially expressed proteins between shCTRL and shGATA4 in neural crest cells. **b** Forty-three proteins were upregulated and 19 proteins were downregulated after GATA4 knockdown in NCCs. **c** Cluster analyses of 1888 gene product ratios measured at the protein level by iTRAQ analysis. Proteins upregulated over the control group are displayed in red, and the downregulated proteins are shown in green. **d** A candidate downregulated protein, BARX1, was examined in NCCs using qRT-PCR (*n* = 5). **e** BARX1 was also examined in pa1 of mice at E10.5 using qRT-PCR (*n* = 5). **f**, **g** Protein expression of BARX1 in the NCCs was tested by Western blotting (*n* = 3). The data are presented as the mean ± S.E.M. from at least three independent experiments. ^*^P < 0.05, ^**^P < 0.01. iTRAQ isobaric tags for relative and absolute quantitation, NCC neural crest cell, qRT-PCR quantitative reverse transcription-polymerase chain reaction, shCTRL short hairpin control (control lentivirus)

Based on the aforementioned in vivo and in vitro results, we sought to examine the potential mechanism underlying the function of GATA4 in NCCs. Thus, iTRAQ analysis was used to identify proteomic changes after GATA4 knockdown in NCCs (Fig. 4a). Among the identified proteins, a group of 43 proteins was significantly upregulated, whereas 19 proteins were significantly downregulated in NCCs after GATA4 knockdown (Fig. 4b, c). Our results revealed that BARX1 was among the downregulated proteins after GATA4 knockdown. BARX1 is widely expressed in the branchial arches [16] and is important for molar [17], cartilage, and jaw development [18]. Because the phenotype of Barx1 mutants [18] is similar to that of GATA4 mutants, we chose BARX1 for further analysis.

In order to verify the proteomic difference observed by iTRAQ analysis, we further measured the expression levels of Barx1 after GATA4 knockdown using quantitative reverse transcription polymerase chain reaction (qRT-PCR) and western blotting assays, respectively. Our results showed that the expression of Barx1 decreased significantly both at mRNA and protein levels after GATA4 knockdown (Fig. 4d–g, Supplementary Figure S2), indicating that Barx1 might function as a potential mediator of GATA4 in NCC development.

### GATA4 binds to the *Barx1* promoter

**Fig. 5:**
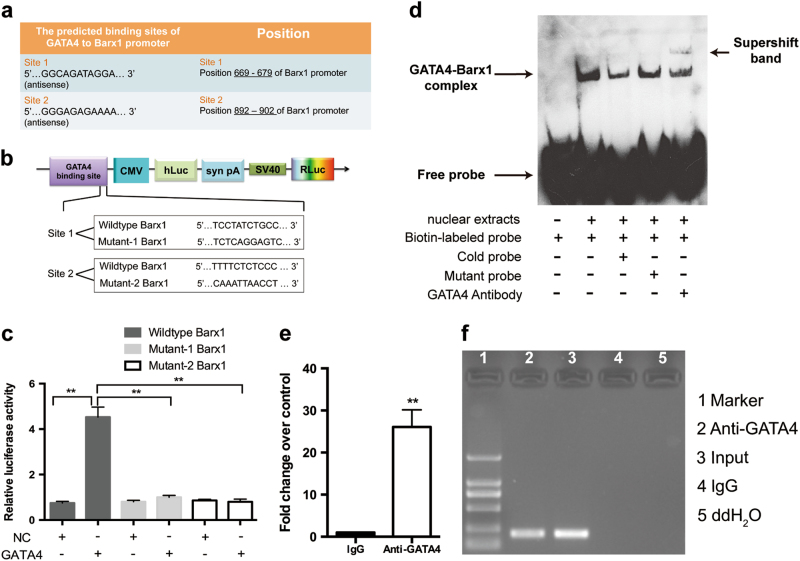
GATA4 is directly associated with the *Barx1* promoter in mice. **a** The information of two binding sites with high scores predicted by computational analysis was chosen for further investigation. The matched sequence of GATA4 to *Barx1* promoter was acquired from the JASPAR database. **b** The upper part corresponds to the structure diagram of the Dual-Luciferase reporter vector (pEZX-FR03). The sequences of the putative GATA4-binding sites in WT and mutants are shown below. **c** Quantitative results of dual-luciferase reporter assay (*n* = 5). “+” or “−” indicated the presence or absence of the reagent in dual-luciferase reporter assay. **d** The nuclear extracts from NCCs were analyzed for binding of GATA4 to *Barx1* promoter using EMSA. Binding of GATA4 to biotin-labeled DNA probes is shown as “GATA4-*Barx1* complex”. “+” or “−” indicated the presence or absence of the reagent in EMSA. The amount of the unlabeled fragment (cold probe and mutant probe) added in the competition assay was 300-folds of the amount of the labeled probe. A supershift band was shown when the nuclear extract was pre-incubated with 2 μg GATA4 antibody, which confirms the presence of GATA4 in both bands. **e**, **f** The quantitative PCR and ChIP gel shift assay for GATA4 binding to *Barx1* promoter in NCCs. Lane 1, DNA marker; Lane 2, ChIP sample with GATA4 antibody; Lane 3, Input amplified by GATA4 primers; Lane 4 ChIP sample with IgG antibody; Lane 5, ddH_2_O amplified by GATA4 primers. In quantitative PCR, *y*-axis indicated fold of enrichment normalized to control immunoglobulin. The data are presented as the mean ± S.E.M. from at least three independent experiments. ^**^P < 0.01. ChIP chromatin immunoprecipitation, ddH_2_O double-distilled water, EMSA electro-mobility shift assay, GAPDH glyceraldehyde 3-phosphate dehydrogenase, IgG immunoglobulin, NCC neural crest cell, WT wild type

Several putative GATA4-binding sites upstream of the transcription start site of the mouse *Barx1* promoter were acquired from the JASPAR database, and two predicted binding sites with high scores were selected for further investigation (Fig. 5a). Both a dual-luciferase assay and electrophoretic mobility shift assay (EMSA) were performed to analyze the respective promoter regions in vitro. In the dual-luciferase assay, we constructed several reporter vectors, including WT GATA4-binding sites or mutant GATA4-binding sites (Fig. 5b). Overexpression of GATA4 enhanced the transcription of *Barx1* compared with that in the controls (Fig. 5c). The mutant GATA4 core binding sequence of the *Barx1* promoter reduced the induction *via* both binding sites (Fig. 5c). As revealed by EMSA, in the first predicted binding site, a retarded band was observed in the lane containing the GATA4-*Barx1* and the biotin-labeled oligonucleotide probe (Fig. 5d) that encompassed the putative GATA4-binding sequence. Since we did not find a binding activity in the second binding site by EMSA assay, we selected the first binding site for further analysis.

To identify direct targets of GATA4 in vivo, a chromatin immunoprecipitation (ChIP) assay was carried out using NCCs. We analyzed the first predicted promoter region and designed primers accordingly. ChIP analysis was performed using anti-GATA4 antibody, and the ChIP input value represented the binding efficiency. The ChIP assay demonstrated a significant increase in the binding efficiency, confirming the in vivo binding of GATA4 to the *Barx1* promoter (Fig. 5e, f).

Full images of EMSA and ChIP assay results are presented in Supplementary Figure S2.

### Knockdown of *gata4* in zebrafish affects NC derivatives

To confirm that *gata4* expression promotes the development of NC derivatives via* barx1*, we adopted a loss-of-function analysis of *gata4* in zebrafish, since both zebrafish embryos and mouse embryos lacking the gene exhibit similar cardiomyopathies [4, 19]. To this end, we examined the phenotype of NC derivatives in zebrafish embryos.

**Fig. 6:**
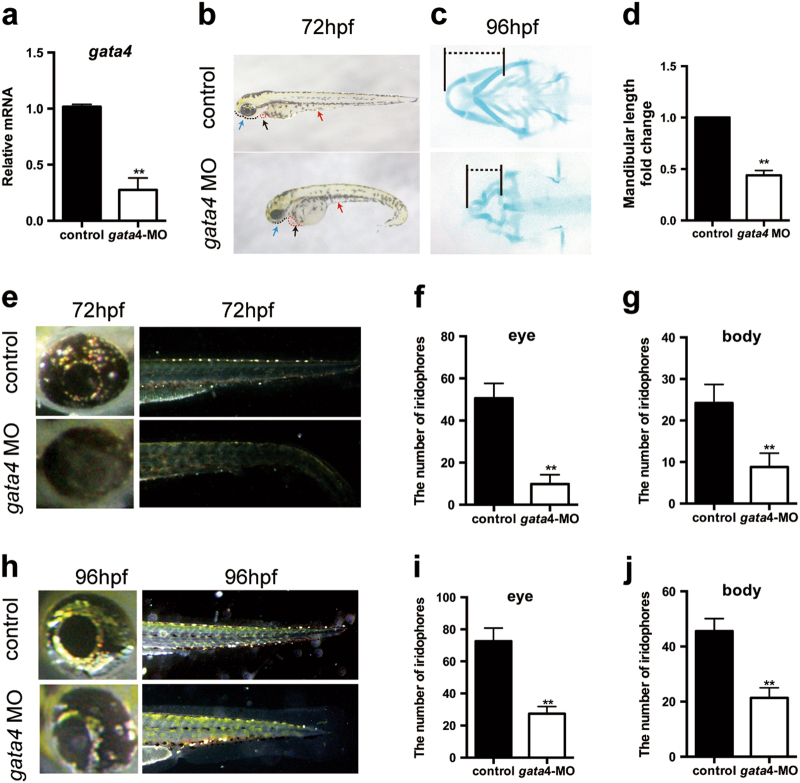
*gata4* is required for the development of the neural crest derivatives in zebrafish. **a** The qRT-PCR analysis showed that *gata4* MO knocked down *gata4* efficiently (*n* = 5). **b** At 72 hpf, zebrafish larvae were imaged with transmitted light. Control embryos and *gata4* MO embryos were injected with a standard control MO and *gata4* MO, respectively. The *gata4* MO embryos showed a shorter body length, mandibular deficiency (black dotted line and blue arrow), edema around the heart (red dotted line and black arrow) and regression of the yolk stalk extension (red arrow). **c** Alcian blue staining revealed a defective mandibular arch compared with controls. The dotted line represents the length of mandible. **d** Quantitative analysis of the length of mandibular arch (*n* = 5). **e** At 72 hpf, the distribution and amount of iridophores were reduced in *gata4* morphants. **f**, **g** Quantitative analysis of the number of iridophores in the eyes and the body (*n* = 5). **h** At 96 hpf, the distribution and amount of iridophores were reduced in *gata4* MO embryos. **i**, **j** Quantitative analysis of the number of iridophores in the eyes and the body (*n* = 5). The data are presented as the mean ± S.E.M. from at least three independent experiments. ^**^P *<* 0.01. hpf hours post-fertilization, MO morpholino, NCC neural crest cell, qRT-PCR quantitative reverse transcription-polymerase chain reaction

To knockdown the expression of *gata4* in zebrafish embryos, we used *gata4* MO. Zebrafish embryos injected with a standard control MO were used as controls. First, we examined the knockdown efficiency of *gata4* MO by qRT-PCR and confirmed that the expression of *gata4* was significantly reduced (Fig. 6a), indicating that *gata4* MO inhibited the function of *gata4*. Subsequently, we observed the morphological defects induced by *gata4* MO at 72 h post fertilization (hpf). Compared with the control embryos, *gata4* MO embryos were smaller and displayed edema around the heart and regression of the yolk stalk extension (Fig. 6b). Moreover, *gata4* MO embryos showed a prominent indentation in the lower jaw (Fig. 6b). Alcian blue staining indicated that the first pair of the cartilaginous pharyngeal arch (mandibular arch) was smaller in *gata4* MO-injected zebrafish at 96 hpf than in controls, with a decrease in length, leading to mandibular retrognathism (Fig. 6c, d). Furthermore, at 72 and 96 hpf, there were fewer iridophores both in the eyes and in the body of *gata4* MO embryos than in controls (Fig. 6e–j). It has been well established that iridophores and the mandibular arch are both NC derivatives. Taken together, these results indicate that *gata4* is required for the normal development of NC derivatives, which is consistent with our results in the mouse model.

### *barx1* mRNA partly restores the defects in *gata4* morphants

**Fig. 7:**
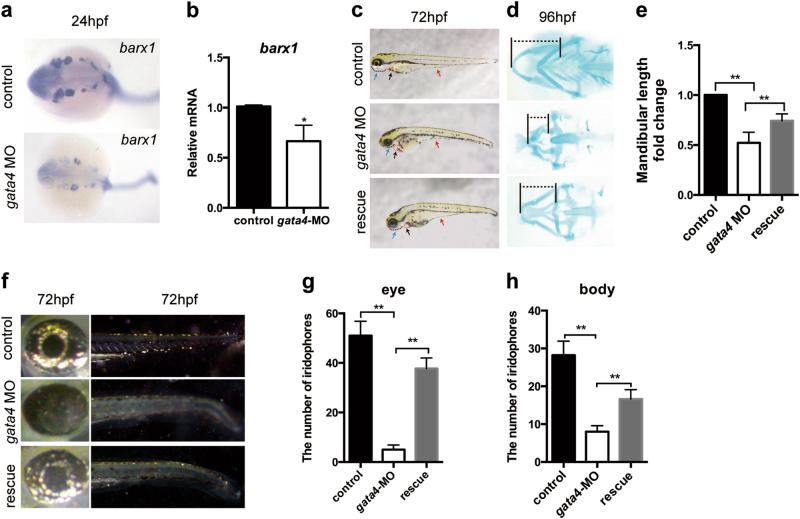
Co-injection of *barx1* mRNA partly rescues the phenotype in *gata4* MO-injected embryos. **a** Embryos underwent in situ hybridization at 24 hpf to detect the expression of *barx1*. **b** Analysis by qRT-PCR to examine the expression of *barx1* after *gata4* knockdown (*n* = 5). **c** At 72 hpf, zebrafish larvae were imaged with transmitted light. Co-injection of *barx1* mRNA partly rescued the mandibular deficiency (black dotted line and blue arrow), edema around the heart (red dotted line and black arrow) and regression of the yolk stalk extension (red arrow). **d** Alcian blue staining showed that co-injection of *barx1* mRNA partly rescues the mandibular deficiency. The dotted line represents the length of mandible. **e** Quantitative analysis of the length of mandibular arch (*n* = 5). **f** The distribution and amount of iridophores were rescued by co-injection of *barx1* mRNA at 72 hpf. **g**, **h** Quantitative analysis of the number of iridophores in the eyes and the body (*n* = 5). The data are presented as the mean ± S.E.M. from at least three independent experiments. ^*^P < 0.05, ^**^P < 0.01. hpf hours post-fertilization, MO morpholino, qRT-PCR quantitative reverse transcription-polymerase chain reaction

*Barx1* is expressed in the pharyngeal arches of both mice and zebrafish. *barx1* mutant zebrafish exhibit a severely reduced lower jaw [18], which is similar to our observation in the *gata4* MO group, indicating that *gata4* may promote the development of NC derivatives *via barx1*. Thus, we examined the expression of *barx1* in zebrafish. Whole-mount in situ hybridization showed that *barx1* expression in the control group at 24 hpf was normal in each of the pharyngeal arches within the dorsal and ventral domains that developed into cartilage elements. However, the *gata4* MO group displayed a significantly lower expression of *barx1* in these areas (Fig. 7a). These results were further confirmed by qRT-PCR analysis, which indicated a nearly 30% decrease in the expression of *barx1* upon the downregulation of *gata4* (Fig. 7b).

To determine whether *barx1* expression was regulated by *gata4*, rescue experiments were carried out by co-injecting *gata4* MO and *barx1* mRNA. Notably, administration of *barx1* mRNA could partially rescue the defects caused by *gata4* deficiency (Fig. 7c). The length of the mandible and the number of the iridophores in the eyes and the body were also partly rescued compared with those in the *gata4* MO group (Fig. 7d–h).

In summary, our findings using two independent animal models indicated that *Barx1* is a downstream effector of GATA4, which thereby trans-activates *Barx1* expression and regulates NC development.

## Discussion

NC developmental defects contribute to clinical pathologies of many human congenital malformations. Understanding the origins of these diseases and the molecular mechanisms governing NC development will help improve current diagnostics and therapeutics. Our study is the first to characterize the relationship between GATA4 and *Barx1* in NC development. Our results demonstrated that GATA4 is essential for normal NC development by promoting NCC proliferation at the developmental stage through direct regulation of *Barx1* at the transcriptional level.

GATA4 has highly conserved zinc finger domains and has been widely studied in the context of early embryonic cardiac development in various species [20, 21, 22]. The structure of the heart is also derived from NCCs. In our study, GATA4 knockout in NCCs resulted in ventricular septal defects and enlarged hearts. The knockdown of *gata4* in our zebrafish model also induced heart failure, which is consistent with previous findings [4]. However, the specific function of GATA4 in NC development has never been studied due to the early lethality in GATA4-deficient mice. Thus, the NCC-specific knockout mouse *Wnt1-Cre;Gata4*^*fl/fl*^ in our study was a useful animal model for studying NC development.

GATA4 was reported to be expressed in migratory NCCs [11], which indicates that it may influence the migration of NCCs. However, in our study, conditional knockout of GATA4 had no effect on the migratory activity of NCCs. Instead, the absence of GATA4 decreased cell proliferation in NCC-derived tissues at developmental time points, potentially leading to the phenotypes of decreased bone mineralization, mandibular retrognathism, and short root deformity of molars. Several studies have revealed positive regulation of GATA4 in the function of osteoblasts [12, 13], which supports our conclusion.

GATA4 as a transcription factor regulates several downstream molecules in different tissues, including the bone morphogenetic protein 4 (*BMP4*) gene, which was identified as a direct downstream target for GATA4 in mesoderm differentiation [23]. However, GATA4 and its downstream molecular events in NC development were still unclear. Through dual-luciferase, EMSA, and ChIP assays, our study indicated that *Barx1* may be one of the direct downstream targets of GATA4 in NCCs. *Barx1* is a homeobox-containing transcription factor that is implicated in the processes of craniofacial, tooth, articular development, and stomach organogenesis [18, 24–27]. Microdeletion of the *Barx1* gene in the human chromosome 9q22.32 is related to craniofacial developmental disorders such as microstomia deformity (small mouth) and mandibular retrusion [28, 29]. Moreover, *barx1* morphants in zebrafish exhibited mandibular arch lengthening [27]. Notably, the phenotypes in humans and zebrafish are similar to the phenotype reported in the present study. This supports our results that *Barx1* may serve as a direct downstream factor of GATA4 involved in NC development.

Taken together, these findings demonstrate that the regulation of GATA4 in NCCs was required for normal NCC development and differentiation. Furthermore, the loss of GATA4 led to NCC-derived craniofacial bone, teeth, and heart hypoplasia characterized by decreased ossification and aberrant cell proliferation. Uncovering the relationship between GATA4 and *Barx1* in NCCs during development is critical to understand the complex morphogenic event. It may also provide direct insight into the causes and potential treatments of similar disorders in humans.

## Materials and Methods

### Animals

*Wnt1-Cre* and *Gata4*^*fl/fl*^ mice have been described elsewhere [9, 19]. To knockout GATA4 specifically in NCCs, we crossed *Wnt1-Cre;Gata4*^*fl/+*^ males with *Gata4*^*fl/fl*^ females (C57/BL6). We used *Gata4*^*fl/fl*^ mice and *Wnt1-Cre* mice as controls and *Wnt1-Cre;Gata4*^*fl/fl*^ as mutants. The mice were obtained from the Model Animal Research Center of Nanjing University (MARC). Zebrafish were raised on a 14/10 h light/dark cycle at 28.5 °C in the zebrafish facility of the Model Animal Research Center, Nanjing University. All experiments were performed with the approval of the Ethics Committee of the Stomatological School of Nanjing Medical University. All procedures were performed according to the guidelines of the Animal Care Committee of Nanjing Medical University.

### Skeletal preparations

According to standard protocols [30], mice were skinned, eviscerated, then fixed in 95% (vol/vol) ethanol, and finally cleared in acetone. Sections were stained with Alcian blue and Alizarin red, and the cartilage and mineralized bone were identified by blue and red staining, respectively. To measure the length of the mouse mandible, the front most part of the mandible and the condylar process were used as the endpoints.

### Micro-CT analysis

Mouse heads were harvested at P21, soft tissues were removed, and the remaining tissues were stored in 70% ethanol overnight. Scanning was performed with a micro-CT scanner (Skyscan, Kontich, Belgium). Slice thickness was 18 μm at 50 kV and 456 μA [31]. Finally, images were reconstructed and analyzed using NRecon v1.6 and CTAn v1.13.8.1 software.

### Histology and immunostaining

*Wnt1-Cre;Gata4*^*fl/fl*^ and *Gata4*^*fl/fl*^ mice were harvested at E9.5, E10.5, E14.5, P1, and P14. Embryos and tissues were carefully dissected and fixed in freshly prepared 4% paraformaldehyde (PFA). The tissue blocks were cut into 5-µm thick sections and mounted on glass slides. Sections were stained with hematoxylin and eosin using standard methods [32]. Von Kossa staining (1% silver nitrate) and total collagen staining (1% Sirius Red) were performed to detect the areas of mineralization [9, 33]. For whole-mount immunostaining, the embryos were blocked with protein block (0.2% bovine serum albumin, 0.2% Triton X-100 in phosphate-buffered saline [PBS]), and stained with rabbit anti-Sox9 (Millipore, #ABE571, 1:1000) [9]. Other antibodies used in this section were rabbit anti-GATA4 antibody (Abcam, #ab84593, 1:300), rabbit anti-collagen I (Abcam, #ab21286, 1:300) and phosphohistone H3 (PHH3; Cell Signaling, #9017, 1:100). The TUNEL assay was performed with the In Situ Cell Death Detection Kit following the manufacturer’s instructions (Roche, #12156792910). Nuclei were stained with 4′,6-diamidino-2-phenylindole (DAPI) at 1:1000. Staining was subsequently visualized by using a laser confocal scanning microscopy (Carl Zeiss, Heidenheim, Germany) and Leica DM 4000 Fluorescence System.

### Culture of NCCs

Embryos were harvested at gestational day E9.5, and deciduas were subsequently removed using microscissors and scalpels. The first branchial arch was extracted in PBS and dissociated in 0.25% trypsin-ethylenediaminetetraacetic acid (Gibco, #25200056) for 30 min at 37 °C, and the dissociated cells were plated into a 6-well dish [34, 35]. Cell suspensions were cultured in Dulbecco’s Modified Eagle Medium: Nutrient Mixture F-12 (1:1) media containing 15% fetal bovine serum supplemented with 100 U/mL penicillin, 100 mg/mL streptomycin, 100 mg/mL L-glutamate, 0.1 mM minimum essential medium non-essential amino acids, 1 mM sodium pyruvate, 55 mM β-mercaptoethanol, 25 ng/mL basic fibroblast growth factor (Gibco, #AA10155), and 1000 U/mL leukemia inhibitory factor (PeproTech, #25002), and then filtered with a 0.22-μm pore size filter.

### Lentiviral transfection

Recombinant lentivirus of shRNA to knockdown *Gata4* expression (shGATA4; 5′-CCAAGCAGGACTCTTGGAA-3′) and control lentivirus (shCTRL; 5′-TTCTCCGAACGTGTCACGT-3′) were purchased from GenePharma (GenePharma, Shanghai, China). The NCCs were seeded in 6-well culture plates at a density of 1.5 × 10^5^ cells per well and cultured overnight. When cells grew to 60–70% confluence, they were infected with the lentivirus (multiplicity of infection = 50) in the presence of 5 μg/mL Polybrene [36]. The medium was replaced with fresh medium after 24 h. The efficiency of the lentiviral infection was measured by fluorescence microscopy 3 days later (Leica Microsystems, Ontario, Canada).

### Apoptosis analysis

For the analysis of cell apoptosis, cells were collected and stained with the Annexin V-FITC/Propidium Iodide Kit (KeyGen Biotech, Nanjing, China) according to the manufacturer’s instructions. NCCs were incubated in the dark for 30 min at room temperature followed by the analysis of the apoptotic cells by flow cytometry [37]. FlowJo V7 software (Tree Star, Oregon, USA) was used to analyze the data.

### Wound scratch assay

The NCCs were transfected with lentivirus and seeded at a density of 1.5 × 10^5^ cells per well in 6-well plates. When cells reached 95–100% confluency, a wound was created using a pipette tip, and the debris and floating cells were subsequently removed by washing the cells once with 0.1 mol/L PBS [38]. Wound closure was captured at 24 h using a Leica DMIRE 2 microscope in phase contrast mode and Leica FW 4000 software.

### CCK8 assay

Transfected cells were seeded into 96-well plates and CCK8 reagent (Dojindo, #CK04) was added to each well for 1 h incubation at 37 °C at five different time points (0, 24, 48, 72, and 96 h). The cell proliferation rate was measured at a wavelength of 450 nm by a microplate reader [39]. Cells infected with a control lentivirus were used as controls.

### Alizarin red staining

Cells were cultured in mineralization-inducing medium for 2 weeks, and the calcification of osteoblast was subsequently analyzed by Alizarin Red staining. After fixation in 10% formaldehyde and rinsing with distilled water, cells were stained with 1% Alizarin Red (Beyotime, Shanghai, China) at room temperature for 20 min [40]. Alizarin red was eluted using 10% cetylpyridinium chloride in 10 mM sodium phosphate for 30 min at room temperature, and the calcium mineral content was subsequently evaluated by measuring the absorbance at 562 nm on a 96-well plate reader.

### Western blotting

Proteins were extracted by using a cell lysis reagent containing the protease inhibitor phenylmethylsulfonyl fluoride [41]. Protein samples were boiled for 5 min and then separated on a denatured SDS-polyacrylamide gel before transfer onto a polyvinylidene difluoride membrane at 300 mA for 1 h. The blotting membrane was blocked with bovine serum albumin for 2 h and then incubated overnight at 4 °C in Barx1 (Abcam, #ab181851, 1:1000) and β-Actin (Santa Cruz Biotechnology, #sc-81178, 1:500). After incubation with secondary antibody at room temperature for 1 h, the membranes were rinsed three times in Tris-buffered saline with Tween-20 and visualized by enhanced chemiluminescence. Semi-quantitative measurements were carried out using ImageJ software.

### Quantitative reverse transcription PCR for mRNA analysis

Total cell RNA was isolated by using TRIzol reagent (Life Technologies, #15596018) according to the manufacturer’s protocol [42]. The mRNA was employed to generate cDNA using a PrimeScript RT reagent kit (TaKaRa, #RR047A). The gene expression level was analyzed by qRT-PCR using the ABI-7300 Real-Time PCR System (Applied Biosystems, CA, USA). The primers used are listed in Supplemental Table S1.

### Protein extraction and iTRAQ labeling

Total proteins of each sample were extracted from the transfected NCCs. After reduction and alkylation by 10 mM dithiothreitol and 55 mM iodoacetamide, each group proteins were digested overnight with trypsin at 37 °C. Finally, samples were labeled with iTRAQ reagents [43]. The samples from both experimental conditions were pooled and subjected to fractionation.

### Plasmid construction

The predicted GATA4-binding site (obtained from the JASPAR database), with the SpeI/XbaI enzyme site and the oligonucleotide sequence (Forward: 5ʹ-CAGCCTCCGGACTCTAGC-3ʹ; Reverse: 5ʹ-TAATACGACTCACTATAGGG-3ʹ) was synthesized and cloned into the SpeI/XbaI site of the pEZX-FR03 luciferase vector after reannealing. The pEZX-FR03 luciferase vector containing the GATA4-binding site was constructed at the same time.

### Dual-luciferase assay

For the luciferase reporter experiments [44], 293T cells were cultured in 24-well plates at a density of 1.0 × 10^5^ cells per well. When cells grew to 80%, they were co-transfected with pEZ-GATA4, pEZ-*Barx1* promoter-luciferase (100 ng per well), and plasmid pEZ-SV40 (10 ng per well) using Polyetherimide. 48 h after transfection, cells were collected and lysed in 1 × passive lysis buffer. Luciferase assays were performed using the Dual-Luciferase Reporter Assay System (Promega, Madison, WI, USA). All samples were tested in triplicate.

### Electro-mobility shift assay (EMSA)

Briefly, nuclear proteins extracted from NCCs were incubated with a biotin-labeled GATA4-binding site DNA probe in binding buffer for 30 min at room temperature. The probe used for the reaction contained the GATA4-binding site of the *Barx1* promoter with a sequence of 5′-CTGAGAGAGGCAGATAGGAAATACA-3ʹ. To determine the binding specificity, “cold probe” or “mutant probe”, which lacked 5ʹ-biotin labeling, were also used in the competitive EMSA to assess the involvement of GATA4 (Supplemental Table S2). A probe lacking nuclear extracts was used as a negative control. The protein–DNA complexes were then electrophoresed on a 6% acrylamide gel and analyzed by autoradiography. Anti-GATA4 polyclonal antibody (0.1 μg/μL; Santa Cruz Biotechnology, #sc-25310 × ) was used for supershift assays. All the experiments were performed in triplicate.

### The ChIP assay

The ChIP assays were performed using EZ-ChIP (Millipore, #17371) according to the manufacturer’s protocol [45]. Briefly, NCCs were incubated in 5 mM dimethyl 3,3-dithiobispropionimidate-HCl (DTBP) for 10 min at room temperature, and cross-linked with 1% formaldehyde for 10 min at 37 °C. Lysates diluted with ChIP dilution buffer were immunoprecipitated with anti-GATA4 (Abcam, #ab86371) or rabbit immunoglobulin as an internal control. Precipitated DNA samples (with or without antibody) as a template were quantified with qPCR to amplify the fragment of *Barx1* promoter. The primers are listed in Supplemental Table S3. Products from qPCR were electrophoresed on 2% agarose gel stained with ethidium bromide and visualized under UV light.

### Microinjection of MO and mRNA

The MO antisense oligonucleotides were synthesized by Gene Tools (Philomath, USA); MO against *gata4* were used [4] as follows: *gata4* MO1 (5ʹ-TCCACAGGTGAGCGATTATTGCTCC-3ʹ), and standard control MO (5ʹ-CCTCTTACCTCAGTTACAATTTATA-3ʹ). All the experiments in this study used 10 ng MO per injection. Total RNA was extracted with Trizol reagent (Ambion) and reverse-transcribed with the Reverse Transcription System (Takara). The sequence of *barx1* cDNA was cloned and ligated to the pGEM plasmid, linearized by BamHI, and transcribed with the mMESSAGE mMACHINE T7 kit (Ambion, #AM1344). The mRNA encoding *barx1* was injected into one-cell stage zebrafish embryos at 0.8 ng/embryo.

### Alcian blue staining

Zebrafish embryos were fixed in 4% PFA at 96 hpf before staining with Alcian blue (Sigma, #A5268) [46]. Embryos were transferred to 30:70 glycerol/1% potassium hydroxide and subsequently to 60:40 glycerol/1% potassium hydroxide and incubated for 2–3 days until they were sufficiently translucent. Specimens were stored and analyzed in 100% glycerol. All the staining procedures were the same in each group. The vertical distance from the beginning to the end of the mandibular arch was used to quantify the mandibular length.

### Whole-mount *in situ* hybridization

Whole-mount *in situ* hybridizations were performed using a standard protocol with modifications [47]. Complementary DNA probes were synthesized using a DIG RNA labeling kit (Roche, #11175025910). The primers listed below were designed by Primer 5.0 software. Whole-mount in situ hybridization of *barx1* was done according to the manufacturer’s protocol. Briefly, embryos at 24 hpf were collected, fixed in 4% PFA overnight at 4 °C. After rinsing three times in PBS at room temperature, embryos were maintained in absolute methanol at −20 or −80 °C for further use. Embryos were rehydrated at gradient dilutions using methanol/PBS (75, 50, and 25%). The embryos were treated with proteinase K (10 μg/mL) at room temperature, refixed with 4% PFA for 20 min, washed three times with PBST (PBS + 0.1% Tween-20), and then prehybridized for 2 h with the hybridization solution without the probe. Subsequently, the digoxin-labeled probe was done according to the standard protocols. The following primers were used (forward/reverse): *barx1* (5ʹ-CACTCTGGTCTCCTCCGT-3ʹ/5ʹ-ATTCTCCGTTTCTCTTCCTC-3ʹ).

### Statistical analysis

All experiments in this study were performed in triplicate to test the reliability of the results. Experimental values were expressed as the mean ± S.E.M. unless otherwise stated. The results in the control and experimental groups were assessed by Student’s *t* test or ANOVA (GraphPad Prism-6 software; San Diego, CA, USA). A *P* value < 0.05 was considered statistically significant.

## Electronic supplementary material

supplementary Information

## References

[1] Yamak A, Latinkic BV, Dali R, Temsah R, Nemer M (2014). Cyclin D2 is a GATA4 cofactor in cardiogenesis. Proc Natl Acad Sci USA.

[2] Zeisberg EM, Ma Q, Juraszek AL, Moses K, Schwartz RJ, Izumo S (2005). Morphogenesis of the right ventricle requires myocardial expression of Gata4. J Clin Invest.

[3] Rivera-Feliciano J, Lee KH, Kong SW, Rajagopal S, Ma Q, Springer Z (2006). Development of heart valves requires Gata4 expression in endothelial-derived cells. Development.

[4] Holtzinger A, Evans T (2005). Gata4 regulates the formation of multiple organs. Development.

[5] Mayor R, Theveneau E (2013). The neural crest. Development.

[6] Etchevers HC, Amiel J, Lyonnet S (2006). Molecular bases of human neurocristopathies. Adv Exp Med Biol.

[7] Jones NC, Lynn ML, Gaudenz K, Sakai D, Aoto K, Rey JP (2008). Prevention of the neurocristopathy Treacher Collins syndrome through inhibition of p53 function. Nat Med.

[8] Keyte A, Hutson MR (2012). The neural crest in cardiac congenital anomalies. Differentiation.

[9] Wiszniak S, Mackenzie FE, Anderson P, Kabbara S, Ruhrberg C, Schwarz Q (2015). Neural crest cell-derived VEGF promotes embryonic jaw extension. Proc Natl Acad Sci USA.

[10] Nelms BL, Labosky PA. Transcriptional control of neural crest development. CA: San Rafael; 2010. 21452438

[11] Pilon N, Raiwet D, Viger RS, Silversides DW (2008). Novel pre- and post-gastrulation expression of Gata4 within cells of the inner cell mass and migratory neural crest cells. Dev Dyn.

[12] Miranda-Carboni GA, Guemes M, Bailey S, Anaya E, Corselli M, Peault B (2011). GATA4 regulates estrogen receptor-alpha-mediated osteoblast transcription. Mol Endocrinol.

[13] Guemes M, Garcia AJ, Rigueur D, Runke S, Wang W, Zhao G (2014). GATA4 is essential for bone mineralization *via* ERα and TGFβ/BMP pathways. J Bone Miner Res.

[14] Narita N, Bielinska M, Wilson DB (1997). Wild-type endoderm abrogates the ventral developmental defects associated with GATA-4 deficiency in the mouse. Dev Biol.

[15] Kuo CT, Morrisey EE, Anandappa R, Sigrist K, Lu MM, Parmacek MS (1997). GATA4 transcription factor is required for ventral morphogenesis and heart tube formation. Genes Dev.

[16] Barlow AJ, Bogardi JP, Ladher R, Francis-West PH (1999). Expression of chick Barx-1 and its differential regulation by FGF-8 and BMP signaling in the maxillary primordia. Dev Dyn.

[17] Miletich I, Buchner G, Sharpe PT (2005). Barx1 and evolutionary changes in feeding. J Anat.

[18] Nichols JT, Pan L, Moens CB, Kimmel CB (2013). barx1 represses joints and promotes cartilage in the craniofacial skeleton. Development.

[19] Watt AJ, Battle MA, Li J, Duncan SA (2004). GATA4 is essential for formation of the proepicardium and regulates cardiogenesis. Proc Natl Acad Sci USA.

[20] Latinkic BV, Kotecha S, Mohun TJ (2003). Induction of cardiomyocytes by GATA4 in Xenopus ectodermal explants. Development.

[21] Epstein JA, Parmacek MS (2005). Recent advances in cardiac development with therapeutic implications for adult cardiovascular disease. Circulation.

[22] Lentjes MH, Niessen HE, Akiyama Y, de Bruine AP, Melotte V, van Engeland M (2016). The emerging role of GATA transcription factors in development and disease. Expert Rev Mol Med.

[23] Nemer G, Nemer M (2003). Transcriptional activation of BMP-4 and regulation of mammalian organogenesis by GATA-4 and -6. Dev Biol.

[24] Tissier-Seta JP, Mucchielli ML, Mark M, Mattei MG, Goridis C, Brunet JF (1995). Barx1, a new mouse homeodomain transcription factor expressed in cranio-facial ectomesenchyme and the stomach. Mech Dev.

[25] Kim BM, Miletich I, Mao J, McMahon AP, Sharpe PA, Shivdasani RA (2007). Independent functions and mechanisms for homeobox gene Barx1 in patterning mouse stomach and spleen. Development.

[26] Mitsiadis TA, Drouin J (2008). Deletion of the Pitx1 genomic locus affects mandibular tooth morphogenesis and expression of the Barx1 and Tbx1 genes. Dev Biol.

[27] Sperber SM, Dawid IB (2008). barx1 is necessary for ectomesenchyme proliferation and osteochondroprogenitor condensation in the zebrafish pharyngeal arches. Dev Biol.

[28] Redon R, Baujat G, Sanlaville D, Le Merrer M, Vekemans M, Munnich A (2006). Interstitial 9q22.3 microdeletion: clinical and molecular characterisation of a newly recognised overgrowth syndrome. Eur J Hum Genet.

[29] Tak HJ, Park TJ, Piao Z, Lee SH (2017). Separate development of the maxilla and mandible is controlled by regional signaling of the maxillomandibular junction during avian development. Dev Dyn.

[30] Chen W, Ma J, Zhu G, Jules J, Wu M, McConnell M (2014). Cbfβ deletion in mice recapitulates cleidocranial dysplasia and reveals multiple functions of Cbfβ required for skeletal development. Proc Natl Acad Sci USA.

[31] Guo S, Ni Y, Ben J, Xia Y, Zhou T, Wang D (2016). Class A Scavenger Receptor Exacerbates Osteoclastogenesis by an Interleukin-6-Mediated Mechanism through ERK and JNK Signaling Pathways. Int J Biol Sci.

[32] Li CY, Hu J, Lu H, Lan J, Du W, Galicia N (2016). αE-catenin inhibits YAP/TAZ activity to regulate signalling centre formation during tooth development. Nat Commun.

[33] Sun W, Sun W, Liu J, Zhou X, Xiao Y, Karaplis A (2010). Alterations in phosphorus, calcium and PTHrP contribute to defects in dental and dental alveolar bone formation in calcium-sensing receptor-deficient mice. Development.

[34] Deng MJ, Jin Y, Shi JN, Lu HB, Liu Y, He DW (2004). Multilineage differentiation of ectomesenchymal cells isolated from the first branchial arch. Tissue Eng.

[35] Ishii M, Arias AC, Liu L, Chen YB, Bronner ME, Maxson RE (2012). A stable cranial neural crest cell line from mouse. Stem Cells Dev.

[36] Zhang H, Wang J, Deng F, Huang E, Yan Z, Wang Z (2015). Canonical Wnt signaling acts synergistically on BMP9-induced osteo/odontoblastic differentiation of stem cells of dental apical papilla (SCAPs). Biomaterials.

[37] Mu W, Hu C, Zhang H, Qu Z, Cen J, Qiu Z (2015). miR-27b synergizes with anticancer drugs *via* p53 activation and CYP1B1 suppression. Cell Res.

[38] Glatzer F, Gschwandtner M, Ehling S, Rossbach K, Janik K, Klos A (2013). Histamine induces proliferation in keratinocytes from patients with atopic dermatitis through the histamine 4 receptor. J Allergy Clin Immunol.

[39] Li QL, Sun YF, Sun YL, Wen JJ, Zhou Y, Bing QM (2014). Mesoporous silica nanoparticles coated by layer-by-layer self-assembly using cucurbit[7]uril for in vitro and in vivo anticancer drug release. Chem Mater.

[40] Yoon H, Kim HL, Chun YS, Shin DH, Lee KH, Shin CS (2014). NAA10 controls osteoblast differentiation and bone formation as a feedback regulator of Runx2. Nat Commun.

[41] Li DF, Liu J, Guo BS, Liang C, Dang L, Lu C (2016). Osteoclast-derived exosomal miR-214-3p inhibits osteoblastic bone formation. Nat Commun.

[42] Ono W, Sakagami N, Nishimori S, Ono N, Kronenberg HM (2016). Parathyroid hormone receptor signalling in osterix-expressing mesenchymal progenitors is essential for tooth root formation. Nat Commun.

[43] O’Brien RN, Shen Z, Tachikawa K, Lee PA, Briggs SP (2010). Quantitative proteome analysis of pluripotent cells by iTRAQ mass tagging reveals post-transcriptional regulation of proteins required for ES cell self-renewal. Mol Cell Proteom.

[44] Shao R, Liu J, Yan G, Zhang J, Han Y, Guo J (2016). Cdh1 regulates craniofacial development *via* APC-dependent ubiquitination and activation of Goosecoid. Cell Res.

[45] Liu WQ, Zhou LY, Zhou CC, Zhang SW, Jing JJ, Xie L (2016). GDF11 decreases bone mass by stimulating osteoclastogenesis and inhibiting osteoblast differentiation. Nat Commun.

[46] de Peralta MSP, Mouguelar VS, Sdrigotti MA, Ishiy FAA, Fanganiello RD, Passos-Bueno MR (2016). Cnbp ameliorates Treacher Collins Syndrome craniofacial anomalies through a pathway that involves redox-responsive genes. Cell Death Dis.

[47] Liu QY, Wu ZL, Lv WJ, Yan YC, Li YP (2007). Developmental expression of Cyclin H and Cdk7 in zebrafish: the essential role of Cyclin H during early embryo development. Cell Res.

