# Supplementary material for: GATA4 as a novel regulator involved in the development of the neural crest and craniofacial skeleton via Barx1
Source: Cell Death Differ. 2018 Mar 9;25(11):1996–2009. doi: 10.1038/s41418-018-0083-x (PMC6219484; doi:10.1038/s41418-018-0083-x)
Supplement: Supplementary file 1 — supplementary Information [file 41418_2018_83_MOESM1_ESM.doc]

## Supplementary Materials and Methods

**Body length measurement**

Measurements of the body length were performed in mice at postnatal day 21 (P21) from the dorsal tip of the nose to the dorsal base of the tail.^1^ The length of tibia was determined by measuring the distance between the proximal and distal articular surfaces.^2^ Total skull length was measured according to a previous study.^3^ The length measurements were based on data from five unique litters.

**Supplementary References**

1. Watanabe Y, Takeuchi K, Higa Onaga S, Sato M, Tsujita M, Abe M*, et al.* Chondroitin sulfate N-acetylgalactosaminyltransferase-1 is required for normal cartilage development. *Biochem J* 2010; **432:** 47–55.

2. Yakar S, Rosen CJ, Beamer WG, Ackert-Bicknell CL, Wu Y, Liu JL*, et al.* Circulating levels of IGF-1 directly regulate bone growth and density. *J Clin Invest* 2002; **110:** 771–781.

3. Vora SR, Camci ED, Cox TC. Postnatal Ontogeny of the Cranial Base and Craniofacial Skeleton in Male C57BL/6J Mice: A Reference Standard for Quantitative Analysis. *Front Physiol* 2015; **6:** 417.


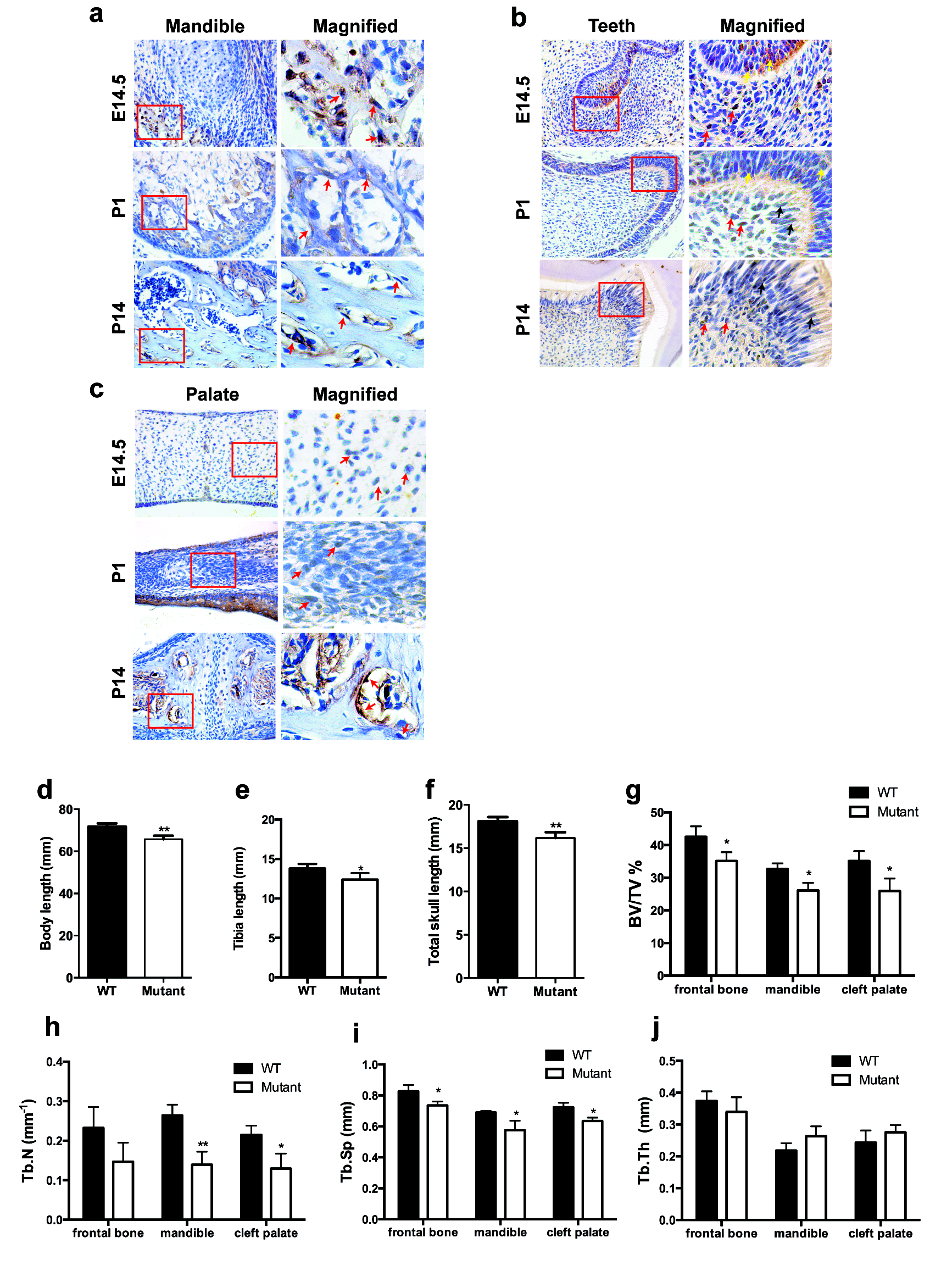


**Supplementary Figure S1. Magnified images from Fig. 1a and measurements of the body structure length and quantification from micro-CT in Fig. 1e, f**

(**a**) Localization of GATA4 expression in the mouse mandible at embryonic day 14.5 (E14.5) and at postnatal day 1 (P1) and P14. The areas within the red boxes are magnified in the images on the right. The red arrows indicate that GATA4 was expressed in the osteoblasts alongside the bone trabecular in the mandible. (**b**) Localization of GATA4 expression in the mouse teeth at embryonic day E14.5, P1 and P14. The areas within the red boxes are magnified in the images on the right. GATA4 was expressed in the dental mesenchyme (red arrows), odontoblasts (black arrows), and ameloblasts (yellow arrows). (**c**) Localization of GATA4 expression in the mouse palate at embryonic day E14.5 (posterior palate section), P1 (posterior palate section) and P14 (posterior palate section). The areas within the red boxes are magnified in the images on the right. At E14.5 and P1, GATA4 was expressed both in the palate epithelium and palate mesenchyme (red arrows). At P14, the GATA4-positive cells (osteoblasts) in the palate were distributed alongside the surface of bone trabecular. (**d–f**) Quantitative analysis of the body length, tibia length, and total skull length, respectively (*n* = 5). (**g–j**) Quantification of micro-CT results shown in Fig. 1e, f. BV/TV, bone volume over total volume; CT, computed tomography; Tb.N, trabecular number; Tb.Sp, trabecular separation; Tb.Th, trabecular thickness; *n* = 3; Data are shown as mean ± S.E.M. *^*^P* < 0.05*, ^**^P* < 0.01


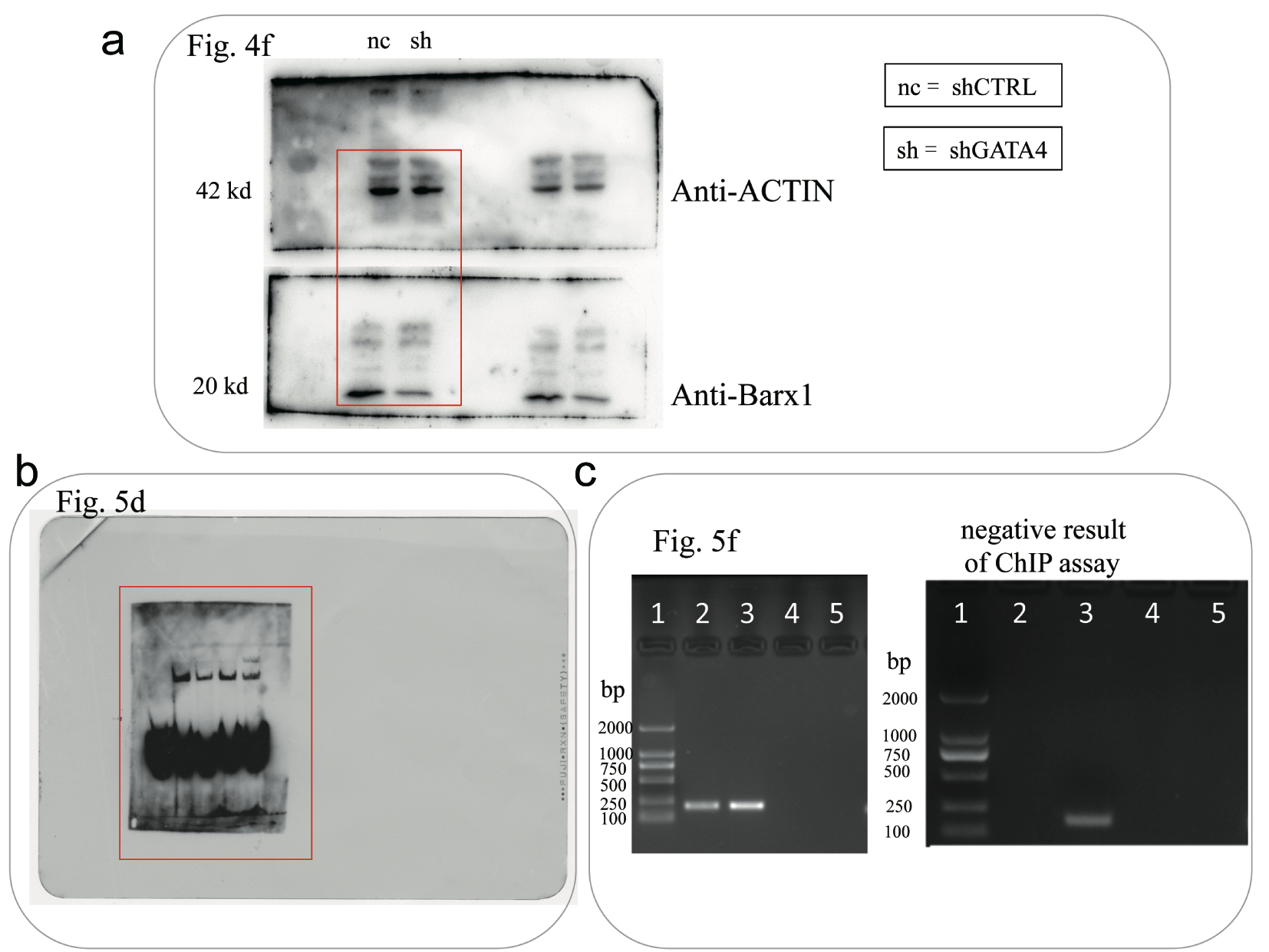


**Supplementary Figure S2. Uncropped images of Western blots, Electrophoretic Mobility Shift Assay (EMSA) results, and more detailed descriptions of chromatin immunoprecipitation (ChIP) assay results.**

(**a**) Uncropped images of Western blots in Figure 4f. (**b**) Uncropped images of EMSA results in Figure 5d. (**c, left**) Image from the ChIP assay in Figure 5f, and the base pair (bp) value of the marker bands are listed in the left. (**c, right**) Image showing the negative result on the ChIP assay in this study. We examined the second binding site in Figure 5a, and the result proves that the anti-GATA4 antibody binding was specific. Lane 1, DNA marker; Lane 2, ChIP sample with GATA4 antibody; Lane 3, Input amplified by GATA4 primers; Lane 4 ChIP sample with IgG antibody; Lane 5, ddH2O amplified by GATA4 primers.

**Supplementary Table S1. Primers for quantitative reverse transcription polymerase chain reaction (qRT-PCR)**

| **Genes** | **Primers** | **Sequences (5′-3′)** |
| --- | --- | --- |
| ***Sox9* (mouse)** | F | AACTGCCTGGAAACTTCTGTGGG |
|  | R | CGGAGGAGGAGGGAGGGAAAA |
| ***Snail2* (mouse)** | F | TCCAAACCCACTCGGATGTGAAGA |
|  | R | TTGGTGCTTGTGGAGCAAGGACAT |
| ***Ets1* (mouse)** | F | AAGTTCTGTATGAGTGGAGCA |
|  | R | CTGGGTAGGTAGGGTTGG |
| ***Msx1* (mouse)** | F | ACCCCTTGCTACACACTTC |
|  | R | GACACTTCCGACCATTCTT |
| ***AP-2* (mouse)** | F | ACCAGCAACGGGACGGCAAGG |
|  | R | TGGCGGAGACAGCATTGCTGTTG |
| ***Twist1* (mouse)** | F | GGAGGATGGAGGGGGCCTGG |
|  | R | TGTGCCCCACGCCCTGATTC |
| ***Nestin* (mouse)** | F | GGACAGGACCAAGAGGAACA |
|  | R | TCCCACCTCTGTTGACTTCC |
| ***Pax3* (mouse)** | F | CTGGTGGTTTCCCTCCCT |
|  | R | TTGGCTGTACTGGTAAGACG |
| ***HNK-1* (mouse)** | F | GCAAGAAGGGCTTCACTGAC |
|  | R | GCCCCCAGAATAGAAAGGAG |
| ***Barx1* (mouse)** | F | AGACAAGGAAGGACACAGG |
|  | R | TCCGAGAAAGACCAGAAG |
| ***Gapdh* (mouse)** | F | TGGATTTGGACGCATTGGTC |
|  | R | TTTGCACTGGTACGTGTTGAT |
| ***β-actin* (zebrafish)** | F | TTCCTTCCTGGGTATGGAATC |
|  | R | GCACTGTGTTGGCATACAGG |
| ***gata4* (zebrafish)** | F | CGGGTGGGTTTATCCT |
|  | R | ATCGCCGACTGACCTT |
| ***barx1* (zebrafish)** | F | ATCCTTTGGAGATTGGGGCG |
|  | R | TAGAAGGTCTCCGGTGGGAC |

**F, forward; R, reverse.**

**Supplementary Table S2. Primers for Electrophoretic Mobility Shift Assay (EMSA)**

| **Genes** | **Primers** | **Sequences (5′-3′)** |
| --- | --- | --- |
| **EMSA-GATA4** | F | GGTTAATTTGGGGAGAGAAAAAGAG |
|  | R | CTCTTTTTCTCTCCCCAAATTAACC |
| **EMSA-GATA4** | F | GGTTAATTTGGGGCGAGAAAAAGAG |
| **(mutant)** | R | CTCTTTTTCTCGCCCCAAATTAACC |

**F, forward; R, reverse.**

**Supplementary Table S3. Primers for chromatin immunoprecipitation (ChIP) assay**

| **Genes** | | **Primers** | | **Sequences (5′-3′)** |
| --- | --- | --- | --- | --- |
| **Barx1 (mouse)** | | F | | GAGAGAGGCAGATAGGAAATACA |
|  | R | | CTGTTGAGTGTGCCTCTTTGA | |

**F, forward; R, reverse.**
